# Effective Gene Silencing in Plants by Synthetic Trans-Acting siRNAs Derived From Minimal Precursors

**DOI:** 10.21769/BioProtoc.5475

**Published:** 2025-10-20

**Authors:** Adriana E. Cisneros, Ana Alarcia, María Juárez-Molina, Alberto Carbonell

**Affiliations:** Instituto de Biología Molecular y Celular de Plantas (Consejo Superior de Investigaciones Científicas–Universitat Politècnica de València), Valencia, Spain

**Keywords:** Plant gene silencing, syn-tasiRNAs, Artificial small RNAs, Transgene-free, Virus-based expression

## Abstract

Synthetic trans-acting small interfering RNAs (syn-tasiRNAs) are 21-nucleotide small RNAs designed to induce highly specific and efficient gene silencing in plants. Traditional approaches rely on the transgenic expression of ~1 kb *TAS* precursors, which limits their use in non-model species, under strict GMO regulations, and in size-constrained expression or delivery systems. This protocol describes a rapid workflow for the design, assembly, and delivery of syn-tasiRNAs derived from much shorter precursors, referred to as minimal precursors. The pipeline includes in silico design of highly specific syn-tasiRNA sequences, cloning of minimal precursors into plant expression or potato virus X (PVX)-based viral vectors through Golden Gate or Gibson assembly, and delivery to plants through *Agrobacterium*-mediated expression or by spraying crude extracts containing recombinant PVX expressing the minimal precursors. These methodologies make syn-tasiRNA-based tools more accessible and broadly applicable for plant research and biotechnology across diverse species and experimental contexts.

Key features

• Syn-tasiRNAs allow the simultaneous silencing of multiple genes with high specificity, as they are computationally designed to avoid off-target effects.

• This protocol describes the design and obtention of syn-tasiRNAs for the simultaneous silencing of one or several endogenous genes in any plant species.

• This protocol also describes a non-transgenic alternative for applying syn-tasiRNAs to plants using a viral vector to induce whole-plant gene silencing.

• This protocol can also be applied to induce antiviral protection against pathogenic viruses, reducing viral mutational escapes when expressing multiple syn-tasiRNAs targeting different viral sites.

## Graphical overview


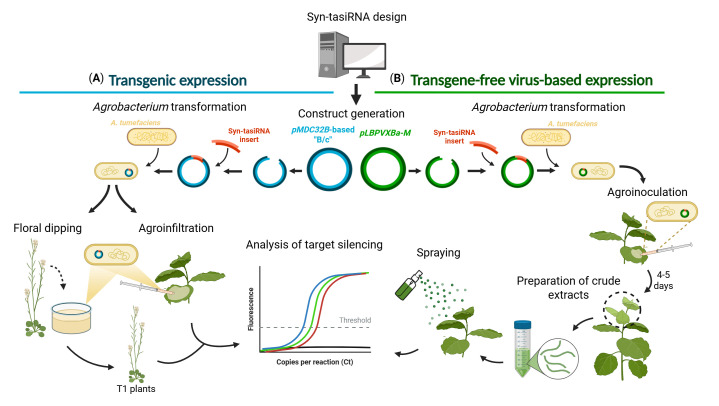


**Steps to generate and apply synthetic *trans*-acting small interfering RNA (syn-tasiRNA) minimal precursors for either transgenic or transgene-free virus-based expression in plants.** The protocol begins with the computational design of syn-tasiRNAs, followed by the generation of the corresponding expression constructs, and concludes with the evaluation of silencing efficacy by real-time quantitative PCR (RT-qPCR) quantification of target mRNA levels. (A) For transgenic expression, the syn-tasiRNA insert is cloned into a *pMDC32B-B/c*-based vector and transformed into *Agrobacterium tumefaciens* for either stable or transient expression. (B) For transgene-free, virus-based expression, the syn-tasiRNA insert is cloned into a Potato virus X (PVX)-based vector and transformed into *A. tumefaciens* for leaf agroinoculation. Once systemic PVX infection is established, upper leaves are collected and homogenized to produce a crude extract, which is then sprayed into a new set of plants to induce whole-plant gene silencing.

## Background

RNA interference (RNAi) is a regulatory mechanism involved in multiple plant biological processes such as stress responses, development, defense, or reproduction [1]. RNAi is characterized by the sequence-specific degradation of RNA molecules mediated by complementary small RNAs (sRNAs). These sRNAs are generated from double-stranded RNA (dsRNA) precursors processed by DICER-Like (DCL) ribonucleases into 20–24 base pair (bp) duplexes. One strand of the duplex is then loaded into an ARGONAUTE (AGO) protein, which guides the complex to sequence-complementary target RNAs, leading to their degradation or translational repression [2]. Initially, several classic RNAi-based tools using long dsRNAs were developed for targeted gene silencing in plants. Although widely used, these strategies lacked high specificity, as DCL-mediated processing of long dsRNAs generates a heterogeneous population of sRNAs that can unintentionally target nonspecific transcripts. To overcome this limitation, a second generation of more specific silencing tools based on 21-nucleotide (nt) artificial sRNAs (art-sRNAs) was developed. Current bioinformatic tools typically design multiple art-sRNA sequences based on the target region and off-target probability, increasing specificity over previous silencing techniques but without completely eliminating off-target risks [3].

**Figure 1.**
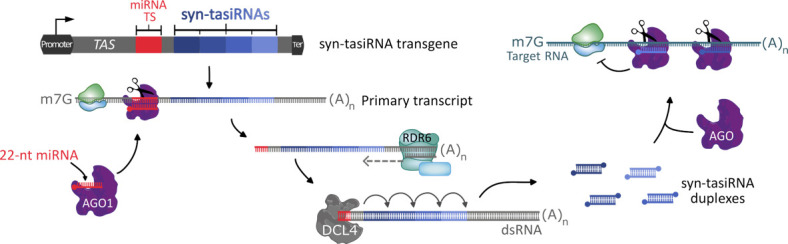
Synthetic *trans*-acting small interfering RNA (syn-tasiRNA) pathway. One or several syn-tasiRNAs are inserted in tandem, replacing the original tasiRNA sequences in a *TAS* precursor. After transcription, the primary transcript is targeted and cleaved by a 22 nucleotide (nt) microRNA (miRNA)–ARGONAUTE1 (AGO1) complex. The RNA-DEPENDENT RNA POLYMERASE 6 (RDR6) synthesizes a double-stranded RNA (dsRNA) from the sliced product, which is then cleaved every 21 nt by DICER LIKE 4 (DCL4), releasing syn-tasiRNA duplexes. Finally, one strand of the duplex is loaded into an AGO protein to silence target RNAs. Ter: terminator. miRNA TS: microRNA target site.

Synthetic *trans*-acting small interfering RNAs (syn-tasiRNAs) are a class of art-sRNAs generated from modified *TAS* transcripts, in which the endogenous tasiRNA sequences are substituted by the designed syn-tasiRNAs. Upon transcription, the modified *TAS* transcript is recognized and cleaved by a miRNA-AGO complex containing a 22-nt endogenous microRNA (miRNA), a critical step that initiates the phase processing and release of the syn-tasiRNAs from the cleaved fragment (Figure 1). Importantly, a single modified *TAS* transcript can produce one or several syn-tasiRNAs, allowing for the simultaneous silencing of multiple genes. Syn-tasiRNAs are typically expressed in plants through *Agrobacterium tumefaciens*–mediated transformation, leading to the generation of transgenic plants, limiting their applications in non-model species or under strict GMO regulations. Furthermore, the considerable size of conventional *TAS* precursors (900–1,000 nt) greatly hampers the use of alternative expression systems like viral vectors.

**Figure 2.**
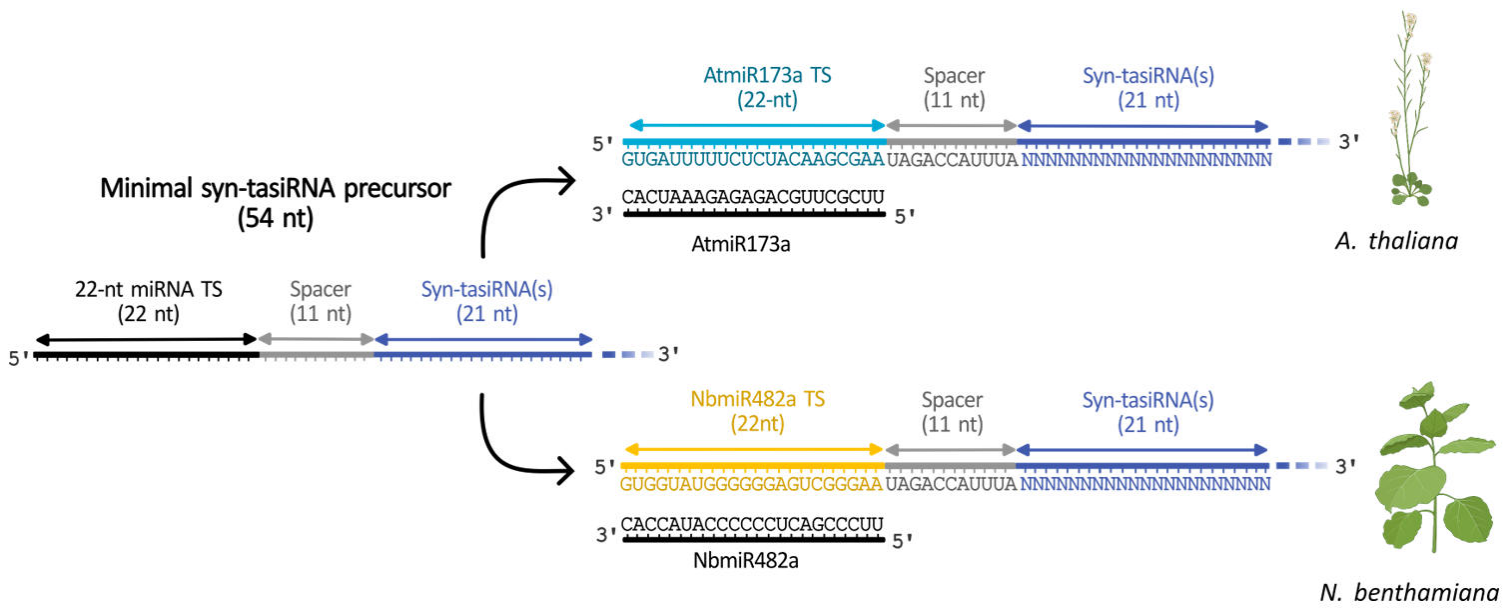
Structure of a minimal synthetic *trans*-acting small interfering RNA (syn-tasiRNA) precursor. The minimal syn-tasiRNA precursor consists of a 22-nucleotide (nt) microRNA target site (miRNA TS), an 11-nt spacer, and a 21-nt syn-tasiRNA sequence. In this example, the precursor expresses a single syn-tasiRNA, but it can be multiplexed to express several syn-tasiRNAs simultaneously. (Top) Minimal precursor containing AtmiR173a TS used to express syn-tasiRNAs in *Arabidopsis thaliana* and closely related species. (Bottom) Minimal precursor containing NbmiR482a TS used to express syn-tasiRNAs in *Nicotiana benthamiana.*

Very recently, a minimal syn-tasiRNA precursor of only 54 nt was developed and proved to be as efficient and accurately processed as canonical *TAS* precursors [4]. This minimal precursor consists of 22-nt from the miRNA target site (TS), an 11-nt spacer, and 21-nt of the syn-tasiRNA, with the possibility of multiplexing if desired (Figure 2). The initial version incorporated AtmiR137a TS for use in *Arabidopsis thaliana (Arabidopsis*), followed by a second variant containing the NbmiR482a TS for *Nicotiana benthamiana* applications (Figure 2). The minimal precursor design enhances cost-efficiency in both in vitro and in vivo production systems for topical delivery applications. Additionally, miRNA TS swapping facilitates adaptation across species, particularly in those expressing an abundant 22-nt miRNA trigger. Importantly, the compact size of the minimal precursor allows its expression from size-restricted systems, such as RNA-based viral vectors, which can only be applied in host species. Furthermore, this approach allows the non-transgenic systemic expression of syn-tasiRNAs following topical application [4]. This protocol describes the steps for the in silico design of highly specific syn-tasiRNAs, the generation of minimal syn-tasiRNA precursors containing either AtmiR137a or NbmiR482aTS, or a customizable TS. Two different application strategies are proposed: i) transgenic expression through genetic transformation, and ii) a transgene-free method based on the exogenous application of Potato virus X (PVX)-based vectors carrying the minimal precursor.

## Materials and reagents

**Biological materials**

1. *pMDC32B-AtmiR173aTS-B/c* (plasmid #227965 Addgene Watertown, MA, USA)

2. *pMDC32B-NbmiR482aTS-B/c* (plasmid #227967; Addgene Watertown, MA, USA)

3. *pMDC32B-B/c* (plasmid #227963; Addgene Watertown, MA, USA)

4. *pLBPVXBa-M* (plasmid #229079; Addgene Watertown, MA, USA)

5. Electrocompetent cells of *Escherichia coli ccd*B-sensitive strain DH5α (homemade, 1 × 10^6^ CFU/μg DNA, see Protocol S1 for details)

6. Electrocompetent cells of *Agrobacterium tumefaciens* strain GV3101 (homemade, 6 × 10^6^ CFU/μg DNA, see Protocol S2 for details)

**Reagents**

1. Liquid nitrogen

2. Sterile Milli-Q water

3. Peat (Kekkilä profesional)

4. Perlite (Projar)

5. GeneArt^TM^ Gibson Assembly HiFi Master Mix (Invitrogen, catalog number: A46627)

*Note: Store at -20 °C, shelf life 1.5 years.*

6. T4 DNA ligase (5 U/μL) (Thermo Fisher Scientific, catalog number: EL0011)

*Note: Store at -20 °C, shelf life 2 years.*

7. Zymo-Spin I columns (Zymo Research, catalog number: C1003-250)

8. GeneJET Plasmid Miniprep kit (Fisher Scientific, catalog number: K0481)

9. Kanamycin monosulfate (Duchefa Biochemie, catalog number: K0126.0005)

*Note: Store at RT, shelf life 5 years.*

10. Rifampicin (Duchefa Biochemie, catalog number: R0146.0005)

*Note: Store at 2–8 °C, light-sensitive, shelf life 4 years.*

11. Agarose (Pronadisa, catalog number: 8016)

12. Red Safe (iNtRON Biotechnology, catalog number: 21141)

13. Agar (INtRON biotechnology, catalog number: 25999)

14. Yeast extract (INtRON biotechnology, catalog number: 48045)

15. Tryptone (INtRON biotechnology, catalog number: 46046)

16. *Bsa*I (10 U/μL, New England Biolabs, catalog number: R3733S)

*Note: Store at -20 °C, shelf life 2 years.*

17. *Mlu*I (10 U/μL) (Thermo Fisher Scientific, catalog number: ER0561)

*Note: Store at -20 °C, shelf life 2 years.*

18. Dithiothreitol (DTT) (from SuperScript^TM^ IV kit, Invitrogen, catalog number: 18091050)

*Note: Store at -20 °C, shelf life 2 years.*

19. NaCl (ITW Reagents, catalog number: 131659.1211)

20. MgCl_2_ (Vidra FOC, catalog number: 131396)

21. CaCl_2_ (PanReac, catalog number: 131232)

22. Na_2_HPO_4_ (PanReac, catalog number: 131678)

23. KH_2_PO_4_ (PanReac, catalog number: 121509)

24. NH_4_Cl (PanReac, catalog number: 131121)

25. MgSO_4_ (PanReac, catalog number: 127113)

26. KCl (PanReac, catalog number: A2939)

27. HCl (PanReac, catalog number: 131020)

28. KOH (PanReac, AppliChem, catalog number: 121515.1211)

29. NaOH (PanReac, catalog number: 131687.1211)

30. Acetosyringone (3’,5’-dimethoxy-4’-hydroxy-acetophenone) (Aldrich, catalog number: D13,440-6)

*Note: Store at RT, shelf life 2 years.*

31. Tris (ITW reagents, catalog number: A1086,1000)

32. EDTA (Sigma, catalog number: E5134-500)

33. Acetic acid glacial (PanReac, catalog number: 131008.1611)

34. Glucose (D-glucose) (Duchefa, catalog number: D0802)

35. Sucrose crystallized (Duchefa Biochemie, catalog number: S0809.1000)

36. 6-benzylaminopurine (BAP) (Duchefa Biochemie, catalog number: B0904.0001)

37. MES (Duchefa, catalog number: M1503)

38. Silwet L-77 (BioWorld, catalog number: 30630216-1)

39. Polyvinylpyrrolidone 10 (PVP) (PanReac AppliChem, catalog number: A2258,0250)

40. Polyethylene glycol 6000 (PEG) (Merck, catalog number: 81260-1KG)

41. 2-mercaptoethanol 14.3 M (BME) (Sigma-Aldrich, catalog number: M6250)

*Note: Store at 2–8 °C, light-sensitive, shelf life 3 years.*

42. Dimethylsulfoxide (DMSO) (Sigma, catalog number: D8418-100 mL)

*Note: Store at RT, shelf life 2 years.*

43. Sodium acetate (3H_2_O) (PanReac AppliChem, catalog number: 131632.1211)

44. Guanidine thiocyanate (PanReac, catalog number: A4335,1000)

45. Ammonium thiocyanate (PanReac, catalog number: 131143.1210)

46. Glycerol 87% (PanReac AppliChem, catalog number: 122329.1211)

47. Phenol 90% (Scharlab, catalog number: FE04791000)

*Note: Store at 4 °C; light-sensitive; shelf life 1 year.*

48. Chloroform (Fisher Scientific, catalog number: 10071970)

*Note: Light-sensitive, shelf life 3 years.*

49. Isopropanol (PanReac AppliChem, catalog number: 1.310.901.214)

50. Ethanol 96% (PanReac AppliChem, catalog number: 141085.1214)

51. TURBO DNA-Free kit (Thermo Fisher, catalog number: AM1907)

*Note: Store at -20 °C.*

52. PrimeScript RT Reagent kit (Takara, catalog number: RR037Q)

*Note: Store at -20 °C.*

53. TB Green Premix Ex Taq (TliRNaseH Plus) (Takara, catalog number: RR420A)

*Note: Long-term storage at -20 °C. Once opened, store at 4 °C and use within 6 months; light-sensitive.*

**Solutions**

1. Soil mix (see Recipes)

2. Liquid Luria Broth (LB) (see Recipes)

3. Solid LB (see Recipes)

4. Kanamycin 50 mg/mL (see Recipes)

5. Rifampicin 50 mg/mL (see Recipes)

6. 1 M KOH (see Recipes)

7. 10 M NaOH (see Recipes)

8. 1 M Tris-HCl pH 7.5 (see Recipes)

9. 1 M MgSO_4_ (see Recipes)

10. 1 M CaCl_2_ (see Recipes)

11. 1 M KCl (see Recipes)

12. 3 M NaCl (see Recipes)

13. 1 M MgCl_2_ (see Recipes)

14. 0.5 M KH_2_PO_4_ pH 8 (see Recipes)

15. Glucose 20% (see Recipes)

16. 3 M sodium acetate pH 5.5 (see Recipes)

17. 1 M MES buffer pH 5.2 (see Recipes)

18. 0.1 M acetosyringone (see Recipes)

19. 0.5 M EDTA pH 8 (see Recipes)

20. Super Optimal broth + catabolic repressor (SOC) (see Recipes)

21. Oligonucleotide annealing buffer (see Recipes)

22. TAE 10× (see Recipes)

23. M9 medium (see Recipes)

24. Vir induction medium (see Recipes)

25. Infiltration solution (see Recipes)

26. Sucrose solution (see Recipes)

27. Inoculation buffer (see Recipes)

28. Saturated phenol (see Recipes)

29. Phenol/guanidine-based extraction buffer (see Recipes)

30. Ethanol 75% (see Recipes)

**Recipes**

**1. Soil mix**

**Table:** 

Reagent	Final concentration	Quantity or volume
Peat	80%	8 kg
Perlite	20%	2 kg

**2. Liquid Luria Broth (LB)**

**Table:** 

Reagent	Final concentration	Quantity or volume
Tryptone	10 g/L	10 g
NaCl	10 g/L	10 g
Yeast extract	5 g/L	5 g
Milli-Q water	n/a	1 L

Divide the final volume into two 500 mL bottles and autoclave. Store at RT.

**3. Solid LB**

**Table:** 

Reagent	Final concentration	Quantity or volume
Tryptone	10 g/L	10 g
NaCl	10 g/L	10 g
Yeast extract	5 g/L	5 g
Milli-Q water	n/a	1 L
Agar	15 g/L	15 g

Divide the final volume into two 500 mL bottles, add 7.5 g of agar and a stir bar to each, and autoclave. Right after sterilizing, add the appropriate amount of antibiotic, mix with a magnetic stirrer, and pour 20–25 mL into each Petri dish, under sterile conditions. Store Petri dishes upside down, covered in plastic film, at 4 °C for up to 2–3 weeks.

**4. Kanamycin 50 mg/mL**

**Table:** 

Reagent	Final concentration	Quantity or volume
Kanamycin	50 mg/mL	1 g
Milli-Q water	n/a	20 mL

Dissolve and sterilize by filtration in a laminar flow cabinet. Store in 1 mL aliquots at -20 °C.

**5. Rifampicin 50 mg/mL**

**Table:** 

Reagent	Final concentration	Quantity or volume
Rifampicin	50 mg/mL	1 g
DMSO	n/a	20 mL

Dissolve and sterilize by filtration in a laminar flow cabinet. Distribute in 1 mL aliquots, cover the tubes in aluminum foil, and store at -20 °C.

**6. 1 M KOH**

**Table:** 

Reagent	Final concentration	Quantity or volume
KOH	1 M	5.6 g
Milli-Q water	n/a	100 mL

Autoclave and store at room temperature (RT).

**7. 10 M NaOH**

**Table:** 

Reagent	Final concentration	Quantity or volume
NaOH	10 M	39.99 g
Milli-Q water	n/a	100 mL

Autoclave and store at RT.

**8. 1 M Tris-HCl pH 7.5**

**Table:** 

Reagent	Final concentration	Quantity or volume
Tris	1 M	12 g
HCl	n/a	6 mL
Milli-Q water	n/a	Up to 100 mL

Dissolve in 80 mL of Milli-Q water and adjust the final pH to 7.5 with HCl. Add Milli-Q water up to the final volume and autoclave. Store at RT.

**9. 1 M MgSO_4_**

**Table:** 

Reagent	Final concentration	Quantity or volume
MgSO_4_	1 M	12 g
Milli-Q water	n/a	100 mL

Autoclave and store at RT.

**10. 1 M CaCl_2_**

**Table:** 

Reagent	Final concentration	Quantity or volume
CaCl_2_	1 M	14.7 g
Milli-Q water	n/a	100 mL

Autoclave and store at RT.

**11. 1 M KCl**

**Table:** 

Reagent	Final concentration	Quantity or volume
KCl	1 M	7.4 g
Milli-Q water	n/a	100 mL

Autoclave and store at RT.

**12. 3 M NaCl**

**Table:** 

Reagent	Final concentration	Quantity or volume
NaCl	3 M	17.5 g
Milli-Q water	n/a	100 mL

Autoclave and store at RT.

**13. 1 M MgCl_2_**

**Table:** 

Reagent	Final concentration	Quantity or volume
MgCl_2_	1 M	50.8 g
Milli-Q water	n/a	250 mL

Autoclave and store at RT.

**14. 0.5 M KH_2_PO_4_ pH 8**

**Table:** 

Reagent	Final concentration	Quantity or volume
KH_2_PO_4_	0.5 M	6.8 g
Milli-Q water	n/a	Up to 100 mL

Dissolve in 80 mL of water, set pH to 8 with 1 M KOH, and add water to the final volume. Autoclave and store at RT.

**15. Glucose 20%**

**Table:** 

Reagent	Final concentration	Quantity or volume
Glucose	20%	20 g
Milli-Q water	n/a	100 mL

Sterilize by filtration and store at RT.

**16. 3 M sodium acetate pH 5.5**

**Table:** 

Reagent	Final concentration	Quantity or volume
Sodium acetate	3 M	40.8 g
Milli-Q water	n/a	Up to 100 mL

Dissolve in 60 mL of Milli-Q water and adjust pH with acetic acid. Add Milli-Q water up to the final volume, autoclave, and store at RT.

**17. 1 M MES buffer pH 5.2**

**Table:** 

Reagent	Final concentration	Quantity or volume
MES	1 M	19.5 g
Milli-Q water	n/a	Up to 100 mL

Add the MES to 50 mL of water and warm up to 37 °C to dissolve. Set the pH to 5.2 with 1 M KOH and add the rest of the water. Sterilize by filtration and store at RT.

**18. 0.1 M acetosyringone**

**Table:** 

Reagent	Final concentration	Quantity or volume
Acetosyringone	0.1 M	0.49 g
DMSO	n/a	25 mL

Sterilize by filtration and store in 1 mL aliquots at -20 °C until use.

**19. 0.5 M EDTA pH 8**

**Table:** 

Reagent	Final concentration	Quantity or volume
EDTA	0.5 M	93 g
Milli-Q water	n/a	Up to 500 mL

Dissolve the EDTA in 200 mL of Milli-Q water and adjust the pH with 10 M NaOH. Heat the solution to completely dissolve EDTA and add Milli-Q water up to the final volume. Autoclave and store at RT.

**20. Super Optimal broth + catabolic repressor (SOC)**

**Table:** 

Reagent	Final concentration	Quantity or volume
Tryptone	20 g/L	20 g
Yeast extract	5 g/L	5 g
NaCl	0.5 g/L	0.5 g
1 M KCl	2.5 mM	2.5 mL
Milli-Q water	n/a	972 mL
Autoclave		
Glucose 20%	0.09%	4.5 mL
1 M MgCl_2_	2.5 mM	2.5 mL

Adjust pH to 7 with 10 M NaOH and add Milli-Q water to the final volume. Distribute into four 250 mL bottles and autoclave. After sterilizing, add the remaining reagents under sterile conditions, distribute in 8 mL aliquots, and store at RT.

**21. Oligonucleotide annealing buffer**

**Table:** 

Reagent	Final concentration	Quantity or volume
1 M Tris-HCl (pH 7.5)	60 mM	60 μL
3 M NaCl	500 mM	166.6 μL
1 M MgCl_2_	60 mM	60 μL
0.1 M DTT	10 mM	100 μL
Sterile Milli-Q water	n/a	Up to 1 mL

Prepare under sterile conditions, distribute in 200 μL aliquots, and store at -20 °C until use.

**22. TAE 10×**

**Table:** 

Reagent	Final concentration	Quantity or volume
Tris	400 mM	48.46 g
0.5 M EDTA (pH 8)	10 mM	20 mL
Acetic acid glacial	200 mM	11.44 mL
Milli-Q water	n/a	Up to 1 L

No need to adjust pH as it should be ~8.5. Autoclave and store at RT. Dilute to 1× prior to use. Store the diluted TAE at RT.

**23. M9 medium**

**Table:** 

Reagent	Final concentration	Quantity or volume
Na_2_HPO_4_	6 g/L	6 g
KH_2_PO_4_	3 g/L	3 g
NaCl	0.5 g/L	0.5 g
NH_4_Cl	1 g/L	1 g
Milli-Q Water	n/a	Up to 1 L

Adjust pH to 5.2 using HCl and add Milli-Q water to the final volume. Autoclave and store at RT.

**24. Vir induction medium**

**Table:** 

Reagent	Final concentration	Quantity or volume
Glucose 20%	11.1 mM	5 mL
1 M MES buffer (pH 5.2)	10 mM	5 mL
0.1 M acetosyringone	0.1 mM	500 μL
1 M CaCl_2_	0.1 mM	50 μL
1 M MgSO_4_	0.5 mM	1 mL
Sterile M9 solution (see Recipe 23)	n/a	500 mL

Prepare under sterile conditions.

**Critical:** Prepare right before use and do not store the leftover solution.

**25. Infiltration solution**

**Table:** 

Reagent	Final concentration	Quantity or volume
1 M MgCl_2_	10 mM	5 mL
1 M MES (pH 5.2)	10 mM	5 mM
0.1 M Acetosyringone	0.15 mM	750 μL
Sterile Milli-Q water	n/a	500 mL

Prepare under sterile conditions.

**Critical:** Prepare right before use and do not store the leftover solution.

**26. Sucrose solution**

**Table:** 

Reagent	Final concentration	Quantity or volume
1 M MgCl_2_	10 mM	10 mL
Sucrose	5%	50 g
BAP	44 mM	10 μL
Milli-Q water	n/a	Up to 1 L

Mix reagents until fully dissolved.

**Critical:** Prepare right before use and do not store the leftover solution.

**27. Inoculation buffer**

**Table:** 

Reagent	Final concentration	Quantity or volume
0.5 M KH_2_PO_4_ (pH 8)	50 mM	10 mL
PVP	1%	1 g
PEG	1%	1 g
Sterile Milli-Q water	n/a	Up to 100 mL
Autoclave		
BME	10 M	0.7 μL/mL inoculation buffer

Autoclave and store at RT.

**Critical:** Add the BME right before use. Do not store the leftover solution after adding BME.

**28. Saturated phenol**

**Table:** 

Reagent	Final concentration	Quantity or volume
Phenol 90%	69.1%	384 mL
Milli-Q water	n/a	116 mL

Mix thoroughly and store at 4 °C in a topaz bottle.

**Caution:** Phenol is toxic if swallowed, in contact with skin, or if inhaled. Prepare the mix with gloves, glasses, and a mask inside a fume hood.

**29. Phenol/guanidine-based extraction buffer**

**Table:** 

Reagent	Final concentration	Quantity or volume
Guanidine thiocyanate	118 g/L	59 g
Ammonium thiocyanate	76 g/L	38 g
3 M Sodium acetate pH 5.5	99.9 mM	16.66 mL
Glycerol 87%	4.9%	28.6 mL
Saturated phenol (Recipe 28)	n/a	190 mL
Milli-Q water	n/a	Up to 500 mL

Dissolve the guanidine thiocyanate and the ammonium thiocyanate in 200 mL of Milli-Q water. Add the rest of the reagents until fully dissolved. Add Milli-Q water up to volume and store at 4 °C in a topaz bottle.

**Caution:** Phenol is toxic if swallowed, in contact with skin, or if inhaled. Prepare the mix with gloves, glasses, and mask inside a fume hood.

**30. Ethanol 75%**

**Table:** 

Reagent	Final concentration	Quantity or volume
Ethanol 96%	75%	78 mL
Milli-Q water	n/a	22 mL

Store at RT.

**Laboratory supplies**

1. Nitrile gloves (StarGuard, catalog number: EcogenSG-N-M-200)

2. Protective coat (LabBox, catalog number: GOSW-00M-001)

3. Protective glasses (Epica S.L., catalog number: 1034)

4. Protective mask (LabBox, catalog number: MASK-002-010)

5. 20 μL pipette (Eppendorf, catalog number: 3123000039)

6. 200 μL pipette (Eppendorf, catalog number: 3123000055)

7. 1,000 μL pipette (Eppendorf, catalog number: 3123000063)

8. 200 μL tubes (UVAT, catalog number: F-0302-01)

9. 1.5 mL tube (SARSTEDT, catalog number: 72690001)

10. 2 mL tube (SARSTEDT, catalog number: 72.691)

11. 2 mL SafeSeal tube (SARSTEDT, catalog number: 72695500)

12. 13 mL tubes (SARSTEDT, catalog number: 60540500)

13. 15 mL conical tubes (SARSTEDT, catalog number: 62.554.502)

14. 50 mL conical tubes (LabBox, catalog number: CTGP-050-050)

15. 96-well PCR plate (SARSTEDT, catalog number: 72.1981)

16. 250 mL glass Erlenmeyer flasks (LabBox, catalog number: EFN3-250-012)

17. 1 L glass Erlenmeyer flasks (LabBox, catalog number: EFN3-1K0-006)

18. 250 mL plastic Beckman tube (Beckman Coulter, catalog number: 356011)

19. Petri dish (SARSTEDT, catalog number: 821473)

20. Round container 20–20 cm wide

21. Mortar and pestle (Porcelaine, LabBox, catalog number: MORK-100-001)

22. Zirconia beads (Biospec Products, catalog number: 11079124zx)

23. Electroporation cuvette (Thermo Fisher, catalog number: 12338212 (5510-11))

24. Spectrophotometer cuvette (Kartell, catalog number: 425-001938)

25. 1 mL needle-less disposable syringes (Vidra FOC, catalog number: 875/01)

26. Miracloth (Millipore; Vidra FOC, catalog number: 475855)

27. 30 mL high-density polyethylene vaporizer (Yizhao)

28. 15 cm pots (Mena Agrícola, catalog number: 750103)

29. Filter 0.2 μm (SARSTEDT, catalog number: 83.1826.001)

30. 100 mL Pyrex bottle (LabBox, catalog number: SBG3-100-001)

31. 500 mL Pyrex bottle (LabBox, catalog number: SBG3-500-001)

32. 500 mL topaz bottle (LabBox, catalog number: SBGA-500-001)

33. Measuring cylinder (Vitlab)

34. Beaker (LabBox, catalog number: BKLP-1K0-006)

35. Spoon (LabBox, catalog number: SPSS-150-005)

## Equipment

1. Millipore Milli-Q Direct 16 Water Purification System

2. Computer connected to the internet

3. Balance (Kern, catalog number: EG2200)

4. Stir bar (lbx, catalog number: MAGC-040-005)

5. Magnetic stirrer (VWR, catalog number: 442-0551)

6. Autoclave (P Selecta)

7. GeneExplorer thermal cycler (Bioer, catalog number: BYQ6617E)

8. Electroporator (Eppendorf, catalog number: 4309000019)

9. Mini Sub Cell GT System with 7 × 10 cm gel tray + mini gel caster + PowerPac supply (Bio-Rad, catalog number: 164-0300)

10. 37 °C walk-in growth chamber

11. 28 °C walk-in growth chamber

12. 37 °C dry bath (Fisher, catalog number: 15387928)

13. 1.5 mL tube centrifuge (Eppendorf, catalog number: 30215964) with rotor (Eppendorf, catalog number: FA-24x2)

14. 50 mL conical tube centrifuge (Eppendorf, catalog number: 5428000205) with rotor (Eppendorf, catalog number: F35-6-30)

15. 250 mL Beckman centrifuge (Avanti J-HC, catalog number: 367502) with rotor (JA-14 Fixed-Angle Aluminum Rotor, 339247)

16. Orbital shaker (VWR, catalog number: 444-0268)

17. Biowave cell density meter wavelength 600 nm (WPA, catalog number: CO8000)

18. Plant walk-in growth chamber

19. Laminar flow cabinet (Telstar Aeolus H6)

20. Fume hood (Flowtronic AFA 1000)

21. pH meter (ViciTac, HACH.LPV200.98.0002)

22. Transilluminator (Cleaver Scientific, model: EZEE geloue)

23. NanoDrop (Thermo Fisher Scientific, model: ND-2000)

24. QuantStudio 3 Real-Time PCR system, 96-well (Thermo Fisher Scientific, catalog number: A28567)

25. Bead beater mixer mill (Retsch, model: MM400)

## Procedure

**A. Syn-tasiRNA insert design**

**A1. Computational design of syn-tasiRNAs**

Highly specific syn-tasiRNAs, including a 5′U nucleotide, a C at position 19, and a mismatch with the target transcript at position 21, can be designed to target endogenous or exogenous genes using the Plant Small RNA Maker Suite (P-SAMS) web app or script (https://github.com/carringtonlab/p-sams), as described before [5]. See General notes 1–5.

**A2. Insert design for plant genetic transformation**

1. Follow the next steps if the target species is either *Arabidopsis* or *N. benthamiana*:

a. For *Arabidopsis* plant transformation, order the *pMDC32B-AtmiR173aTS-B/c* from Addgene. For *N. benthamiana* plant transformation, order the *pMDC32B-NbmiR482aTS-B/c* vector from Addgene.

b. Order the following oligonucleotides containing the previously designed syn-tasiRNAs (2, as in Table 1):

**Table 1.** Oligonucleotides for syn-tasiRNA minimal precursor expression from *pMDC32B-AtmiR173aTS-B/c* or *pMDC32B-NbmiR482aTS-B/c*

Oligonucleotide	5′ to 3′ DNA sequence
Forward	TTTAX_1_X_2_X_3_X_4_X_5_X_6_X_7_X_8_X_9_X_10_X_11_X_12_X_13_X_14_X_15_X_16_X_17_X_18_X_19_X_20_X_21_ **X_1_X_2_X_3_X_4_X_5_X_6_X_7_X_8_X_9_X_10_X_11_X_12_X_13_X_14_X_15_X_16_X_17_X_18_X_19_X_20_X_21_**
Reverse	CCGA**Y_21_Y_20_Y_19_Y_18_Y_17_Y_16_Y_15_Y_14_Y_13_Y_12_Y_11_Y_10_Y_9_Y_8_Y_7_Y_6_Y_5_Y_4_Y_3_Y_2_Y_1_** Y_21_Y_20_Y_19_Y_18_Y_17_Y_16_Y_15_Y_14_Y_13_Y_12_Y_11_Y_10_Y_9_Y_8_Y_7_Y_6_Y_5_Y_4_Y_3_Y_2_Y_1_

X_1_–X_21_ is the syn-tasiRNA-1 sequence.

**X_1_–X_21_** is the syn-tasiRNA-2 sequence.

**Y_21_–Y_1_** is the reverse complement of syn-tasiRNA-2

Y_21_–Y_1_ is the reverse complement of syn-tasiRNA-1.

**Figure 3.**
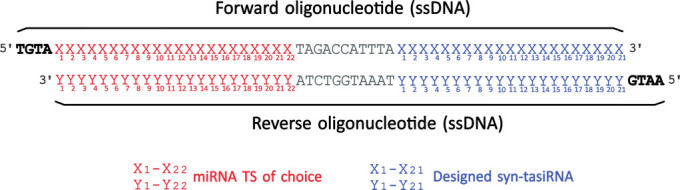
Design of synthetic *trans-*acting small interfering RNA (syn-tasiRNA) minimal precursor insert for *pMDC32B-B/c-*based construct generation. Complementary single-stranded DNA (ssDNA) oligonucleotides are designed to include a 22-nucleotide (nt) microRNA target site (miRNA TS) of choice (red “X” and “Y”), an 11-nt spacer (grey), and a 21-nt syn-tasiRNA sequence (blue “X” and “Y”; one in this example). These oligonucleotides are annealed to form the minimal precursor insert, flanked by 5′-TGTA and 5′-AATG overhangs (bold), compatible by Golden Gate assembly with *Bsa*I-linearized *pMDC32B-B/c.*

2. Follow the next steps in the case of using a 22-nt miRNA TS of choice:

a. Order the *pMDC32B-B/c* vector from Addgene.

b. Order the following oligonucleotides containing the 22-nt miRNA TS of interest and the previously designed syn-tasiRNAs (Figure 3; 2 as in Table 2):

**Table 2.** Oligonucleotides for syn-tasiRNA minimal precursor expression from *pMDC32B-B/c*

Oligonucleotide	5′ to 3′ DNA sequence
Forward	TGTAX_1_X_2_X_3_X_4_X_5_X_6_X_7_X_8_X_9_X_10_X_11_X_12_X_13_X_14_X_15_X_16_X_17_X_18_X_19_X_20_X_21_X_22_ TAGACCATTTA X_1_X_2_X_3_X_4_X_5_X_6_X_7_X_8_X_9_X_10_X_11_X_12_X_13_X_14_X_15_X_16_X_17_X_18_X_19_X_20_X_21_ **X_1_X_2_X_3_X_4_X_5_X_6_X_7_X_8_X_9_X_10_X_11_X_12_X_13_X_14_X_15_X_16_X_17_X_18_X_19_X_20_X_21_**
Reverse	AATG**Y_21_Y_20_Y_19_Y_18_Y_17_Y_16_Y_15_Y_14_Y_13_Y_12_Y_11_Y_10_Y_9_Y_8_Y_7_Y_6_Y_5_Y_4_Y_3_Y_2_Y_1_** Y_21_Y_20_Y_19_Y_18_Y_17_Y_16_Y_15_Y_14_Y_13_Y_12_Y_11_Y_10_Y_9_Y_8_Y_7_Y_6_Y_5_Y_4_Y_3_Y_2_Y_1_TAAATGGTCTAY_22_Y_21_Y_20_Y_19_Y_18_Y_17_Y_16_Y_15_Y_14_Y_13_Y_12_Y_11_Y_10_Y_9_Y_8_Y_7_Y_6_Y_5_Y_4_Y_3_Y_2_Y_1_

X_1_–X_22_ is the 22-nt miRNA TS.

X_1_–X_21_ is the syn-tasiRNA-1 sequence.

**X_1_–X_21_** is the syn-tasiRNA-2 sequence.

**Y_21_–Y_1_** is the reverse complement of syn-tasiRNA-2.

Y_21_–Y_1_ is the reverse complement of syn-tasiRNA-1.

Y_22_–Y_1_ is the reverse complement of the 22-nt miRNA TS.

**A3. Insert design for transgene-free virus-based expression**

**Figure 4.**
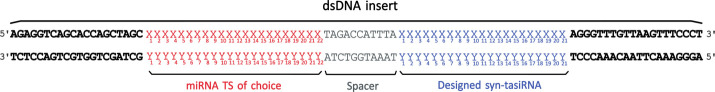
Design of synthetic *trans-*acting small interfering RNA (syn-tasiRNA) minimal precursor insert for *pLBPVXBa-M-*based construct generation. Double-stranded DNA (dsDNA) insert is composed of a minimal syn-tasiRNA precursor including 22-nucleotide (nt) microRNA target site (miRNA TS) of choice (red “X” and “Y”), an 11-nt spacer (grey), and a 21-nt syn-tasiRNA sequence (blue “X” and “Y”; one in this example). Moreover, the insert also contains 20 base pairs flanking extensions (bold), compatible by Gibson assembly with *Mlu*I-linearized *pLBPVXBa-M.*

1. Follow the next steps to design a syn-tasiRNA-based insert for minimal precursor expression from a PVX-based vector.

a. Order the *pLBPVXBa-M* vector from Addgene.

b. Order a dsDNA molecule as an ultramer with the following sequence, including the 22-nt miRNA of choice and the previously designed syn-tasiRNAs (Figure 4; 2 in the following example):

agaggtcagcaccagctagcX_1_X_2_X_3_X_4_X_5_X_6_X_7_X_8_X_9_X_10_X_11_X_12_X_13_X_14_X_15_X_16_X_17_X_18_X_19_X_20_X_21_X_22_ TAGACCATTTAX_1_X_2_X_3_X_4_X_5_X_6_X_7_X_8_X_9_X_10_X_11_X_12_X_13_X_14_X_15_X_16_X_17_X_18_X_19_X_20_X_21_ **X_1_X_2_X_3_X_4_X_5_X_6_X_7_X_8_X_9_X_10_X_11_X_12_X_13_X_14_X_15_X_16_X_17_X_18_X_19_X_20_X_21_** agggtttgttaagtttccct

X_1_-X_22_ is the 22-nt miRNA TS.

X_1_-X_21_ is the syn-tasiRNA-1 sequence.

**X_1_-X_21_** is the syn-tasiRNA-2 sequence.

x is the tail complementary to the PVX sequence and required for the Gibson-based assembly.

See General note 6.

**B. Syn-tasiRNA construct generation**

**Figure 5.**
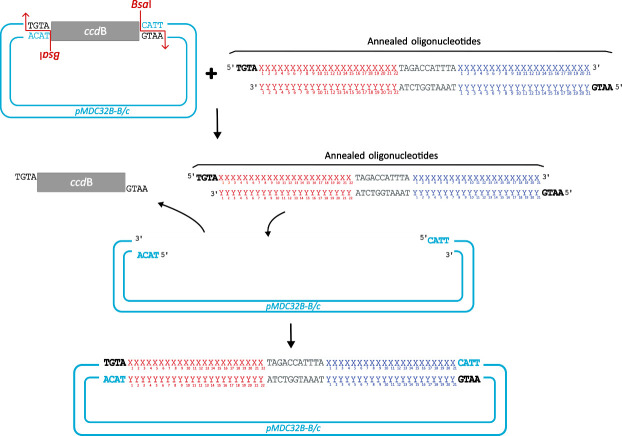
Steps for cloning minimal synthetic *trans*-acting small interfering RNA (syn-tasiRNA) precursors into *pMDC32B-B/c* vectors through Golden Gate assembly. The insert is generated by annealing two complementary DNA oligonucleotides (detailed in Figure 3) containing a microRNA target site (red “X” and “Y”), a spacer (grey), and the syn-tasiRNA of interest (blue “X” and “Y”; one in this example). The *pMDC32B-B/c* vector contains a *ccd*B cassette (grey square) flanked by two *Bsa*I recognition sites (indicated by red arrows). During the digestion-ligation reaction, the *ccd*B cassette is excised by *Bsa*I, and the insert, with compatible 5′ overhangs (bold), is ligated into the vector.

1. For syn-tasiRNA cloning into *pMDC32B-*based “B/c” vectors through Golden Gate assembly for **plant transformation**, follow the next steps (Figure 5).

a. Resuspend the two oligonucleotides in sterile Milli-Q water to a final concentration of 100 μM.

b. Assemble the oligonucleotide annealing reaction in a 200 μL tube as described in Table 3.

**Table 3.** Oligonucleotide annealing mix

Reagent	Volume
Forward oligonucleotide (100 μM)	2 μL
Reverse oligonucleotide (100 μM)	2 μL
Oligo annealing buffer (see Recipes)	46 μL

c. Transfer the tube to a thermocycler set to heat the annealing reaction for 5 min at 94 °C and then cool down to 20 °C at a rate of 0.05 °C/s.

*Note: Alternatively, the annealing reaction can be done in a water bath or a thermoblock by heating for 5 min at 94 °C and then turning off the apparatus. Let the reaction cool down until it reaches RT.*

d. To perform the digestion-ligation reaction with a balanced insert/vector ratio, dilute the annealed oligonucleotides to a final concentration of 0.15 μM by adding 3 μL of the annealed oligonucleotides to 37 μL of sterile Milli-Q water.

**Critical:** Do not store diluted oligonucleotides.

e. Assemble the digestion-ligation reaction as described in Table 4.

**Table 4.** Digestion-ligation reaction mix

Reagent	Volume
*pMDC32B-*based vector (50 ng/μL)	1 μL
Diluted annealed oligonucleotides (0.15 μM)	1 μL
10× T4 DNA ligase buffer	1 μL
T4 DNA ligase (5 U/μL)	1 μL
*Bsa*I (10 U/μL)	1 μL
Sterile Milli-Q water	Up to 10 μL

f. Mix the reactions by pipetting and incubate for 5 min at 37 °C.

*Note: The incubation time of the digestion-ligation reaction can be increased up to 30 min if necessary.*

**Pause point:** Reactions can be stored at 4 °C until use.

g. Use 1–5 μL of the digestion-ligation to electroporate a *ccd*B-sensitive *E. coli* strain. Plate all the culture in a solid LB (see Recipes) Petri dish containing kanamycin 50 μg/mL (final concentration). Leave at 37 °C overnight. See Troubleshooting 1.

h. Use a sterile 1 μL pipette tip to pick two colonies per construct and grow in 4 mL of liquid LB (see Recipes) with kanamycin (50 μg/mL final concentration) in a 13 mL tube at 37 °C overnight on a shaker incubator.

i. Purify plasmids from the grown culture with a Miniprep kit.

j. Digestion of good clones with *Xba*I yields two bands of 9,970 and 225 bp.

k. Confirm clone identity by Sanger sequencing with the following oligonucleotides (Table 5):

**Table 5.** attB1 and attB2 oligonucleotide sequences

Oligonucleotide	5′ to 3′ DNA sequence
AC-285	ACAAGTTTGTACAAAAAAGCAGGCT
AC-286	ACCACTTTGTACAAGAAAGCTGGGT

*Note: If cloning a single syn-tasiRNA, positive clones will include the 54-bp insert (containing the miRNA TS of interest, a spacer, and the designed syn-tasiRNA), located 29 bp downstream of the forward oligonucleotide (AC-285) binding site. Sequencing with the reverse AC-286 oligonucleotide can be done to confirm the sequence.*

**Figure 6.**
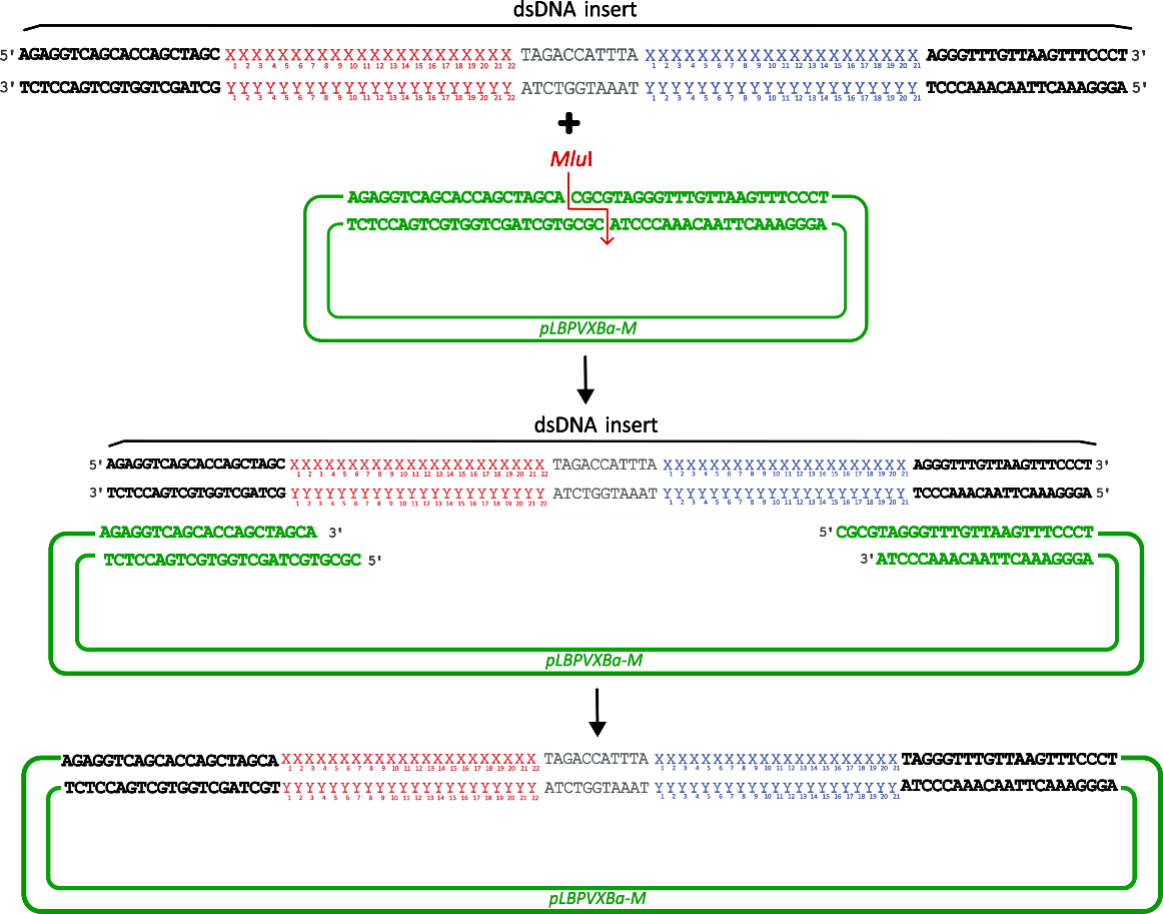
Steps for cloning minimal synthetic trans-acting small interfering RNA (syn-tasiRNA) precursors into the pLBPVXa-M vector through Gibson assembly. The insert is a double stranded DNA (dsDNA) molecule (detailed in Figure 4) containing a microRNA target site (red “X” and “Y”), a spacer (grey), and the syn-tasiRNA of interest (blue “X” and “Y”; one in this example), flanked by 20-base pair extensions at both ends (black bold). These extensions are compatible with the MluI-digested pLBPVXa-M vector (green bold), allowing integration through Gibson assembly.

2. To clone syn-tasiRNA into *pLBPVXBa-M* through Gibson assembly, for viral vector-mediated expression and subsequent generation of **non-transgenic plants**, follow these steps (Figure 6):

a. Add the corresponding amount of sterile water to each duplexed ultramer oligonucleotide to get a final concentration of 1 μg/μL.

b. Digest an aliquot of *pLBPVXBa-M* with *Mlu*I as described in Table 6.

**Table 6.** *Mlu*I digestion reaction mix

Reagent	Volume
*pLBPVXBa-M* (50–100 ng/μL)	10 μL
*Mlu*I (10 U/μL)	0.5 μL
10× R buffer	2 μL
Sterile Milli-Q water	7.5 μL

c. Set at 37 °C for at least 1 h. See Troubleshooting 2.

d. Gel purify the 9,934 bp band corresponding to the linearized plasmid.

e. Assemble the Gibson reaction in a 200 μL tube as described in Table 7.

**Table 7.** Gibson assembly reaction mix

Reagent	Volume
Linearized *pLBPVXBa-M*	^a^
Resuspended duplexed ultramer	^b^
GeneArt Gibson Assembly HiFI Master Mix	5 μL
Sterile Milli-Q water	Up to 10 μL

^a^The optimal volume of the linearized vector is 50–100 ng.

^b^Add the proper volume of insert for an insert/vector molar ratio of 5 since the insert is less than 200 bp.

**Critical:** Total DNA amount must be within the range of 0.02–0.5 pmol.

*Note: Mass to mole conversion can be calculated* *here* .

f. Incubate reactions at 50 °C for 1 h in a thermocycler. Purify the reactions with a DNA-binding spin column like Zymo-spin columns.

g. Use 1–4 μL of the cleaned reaction to electroporate *E. coli* DH5α cells. Plate all the culture in a solid LB (see Recipes) Petr dish containing kanamycin (50 μg/mL final concentration). Leave at 37 °C overnight.

h. Use a sterile 1 μL pipette tip to pick two colonies per construct and grow in 4 mL of liquid LB (see Recipes) with kanamycin (50 μg/mL final concentration) in a 13 mL tube at 37 °C overnight on a shaking incubator.

i. Purify plasmids from the grown culture with a Miniprep kit.

j. Digestion of good clones with *Apa*I and *Xho*I yields two bands of 8,595 and 1,374 bp.

k. Confirm clone identity by Sanger sequencing with the following oligonucleotides (Table 8):

**Table 8.** Oligonucleotides for *pLBPVXBa-*based construct confirmation

Oligonucleotide	5′ to 3′ DNA sequence
Forward (AC-654)	GGGAATCAATCACAGTGTTGGC
Reverse (AC-655)	GCTACTATGGCACGGGCTGTAC

*Note: If cloning a single syn-tasiRNA, good clones will include the 54-bp insert (containing the miRNA TS of interest, a spacer, and the designed syn-tasiRNA), located 108 bp downstream of the forward oligonucleotide (AC-654) binding site. Sequencing with the reverse AC-655 oligonucleotide can be done to confirm the sequence. See Troubleshooting 3.*

**C. *Agrobacterium tumefaciens* transformation**

1. Use 0.5 μL of each purified plasmid to electroporate *A. tumefaciens* GV3101 cells. Plate 1/10 of the culture in a solid LB Petri dish containing rifampicin and kanamycin (50 μg/mL final concentration).

2. Incubate the plate at 28 °C for 48 h.

3. Store the plate with grown colonies at 4 °C until use.

**D. Transgenic expression of syn-tasiRNAs (Option I)**

See General note 7.

**1. Agroinfiltration for transient expression in *N. benthamiana***

a. From a fresh plate, use a sterile 1 μL pipette tip to pick a single colony of *A. tumefaciens* transformed with the corresponding *pMDC32B*-based syn-tasiRNA construct and transfer to a 13 mL tube containing 5 mL of liquid LB with rifampicin and kanamycin (50 μg/mL final concentration).

b. Incubate the starter culture at 28 °C for 24 h on a shaker incubator (200 rpm). See Troubleshooting 4.

c. Transfer 4 mL of the starter culture to 50 mL of liquid LB containing kanamycin (50 μg/mL final concentration) in 250 mL flasks.

d. Incubate on a shaker incubator (200 rpm) at 28 °C for 4–6 h until the culture optical density at 600 nm (OD_600_) reaches ~0.5.

e. Transfer 45 mL of the culture to a 50 mL conical tube and centrifuge in an Eppendorf F35-6-30 rotor (brake ON) for 10 min at 3,354× *g*.

f. Remove the supernatant and resuspend the pellet in 45 mL of Vir induction medium (see Recipes) by gentle shaking. Do not add antibiotics to Vir induction medium.

g. Transfer back to 250 mL flasks and leave at 28 °C on a shaker incubator (200 rpm) overnight.

h. Initial OD_600_ after overnight incubation should be ~1.1–1.2. After measuring the OD_600_ of all cultures, calculate the centrifugation volume for a final volume of 15 mL at a final OD_600_ of ~1.1.

i. Transfer the calculated volume to a 50 mL conical tube and centrifuge in an Eppendorf F35-6-30 rotor (brake ON) for 10 min at 2,795× *g*.

j. Remove the supernatant and resuspend the pellet in 15 mL of infiltration solution (see Recipes) by gentle shaking.

k. Measure the OD_600_ and normalize cultures to a final OD_600_ 1.0.

*Note: 0.8 is also an acceptable OD_600_.*

l. Mix each syn-tasiRNA culture with an equal volume of *A. tumefaciens* culture including an empty vector or control construct such as *pMDC32-GUS* [6].

**Caution:** Use protective glasses to infiltrate.

m. Infiltrate the whole surface of the fourth and fifth leaves (counting from the bottom) of 3–4-week-old *N. benthamiana* plants. See General notes 8 and 9. See Troubleshooting 5.

**2. Total RNA isolation from *N. benthamiana* leaves**

a. Typically 2–3 days post-agroinfiltration, collect and combine the two infiltrated leaves from each individual plant.

**Caution:** Liquid nitrogen can cause severe cryogenic burns; manipulate with protective gloves and glasses.

b. Snap-freeze leaves in liquid nitrogen.

**Pause point:** Frozen leaves can be stored at -80 °C until use.

**Critical:** Tissue must remain frozen throughout the grinding process. Pre-chilling the mortar and pestle with liquid nitrogen before placing the tissue helps prevent sample from thawing.

c. Place the leaves in a porcelain mortar and pour 30–50 mL of liquid nitrogen.

**Critical:** Perform the following steps on ice.

**Caution:** The following steps involve using hazardous reagents; use protective gloves and glasses and work in a fume hood.

d. Lightly crush leaves after liquid nitrogen has completely evaporated; grind vigorously until tissue is powdered.

e. Add 5 mL of phenol/guanidine-based extraction buffer (see Recipes) and continue homogenization to mix the partially frozen sample.

f. Transfer the homogenized sample to a pre-chilled 15 mL plastic conical tube.

g. Centrifuge at 4 °C for 10 min in an Eppendorf F35-6-30 rotor (brake ON, using 15 mL tube adapters) at 5,478× *g* to pellet cell debris.

h. Pour off the supernatant to a new pre-chilled 15 mL plastic conical tube.

i. Add 3 mL of cold chloroform, close tubes tightly, and shake vigorously for 20 s.

j. Incubate for 3 min at RT.

k. Centrifuge at 4 °C for 10 min in an Eppendorf F35-6-30 rotor (brake ON, using 15 mL tube adapters) at 5,478× *g* to separate phases.

**Critical:** Handle tubes gently to avoid mixing separated phases.

l. Use a 1,000 μL pipette to carefully transfer the aqueous (upper) phase to a new pre-chilled 15 mL plastic conical tube.

m. Add 5 mL of cold isopropanol and close tubes.

n. Invert 5–10 times and incubate for 10 min at RT.

o. Centrifuge at 4 °C for 10 min in an Eppendorf F35-6-30 rotor (brake ON, using 15 mL tube adapters) at 11,180× *g* to pellet total RNA.

p. Pour off supernatant and wash the pellet with 1 mL of cold 75% ethanol (see Recipes) by adding and immediately removing with a pipette.

q. Air-dry for 2 min and remove the residual ethanol with a pipette.

r. Air-dry for 2 min and add 100–200 μL of sterile Milli-Q water. Resuspend the pellet by pipetting up and down.

s. Transfer to a pre-chilled 1.5 mL tube and quantify RNA concentration with a NanoDrop.

t. Store at -80 °C until use.

**3. Floral dipping for stable expression in *Arabidopsis***

See General note 10.

a. In the morning, from a fresh plate, using a sterile 1 μL pipette tip, pick two isolated colonies of each *A. tumefaciens* transformed with the corresponding *pMDC32B*-based syn-tasiRNA construct. Transfer to a 13 mL tube containing 5 mL of liquid LB with rifampicin (100 μg/mL final concentration) and kanamycin (50 μg/mL final concentration).

b. Incubate the starter culture at 28 °C for 24 h on a shaker incubator at 200 rpm.

c. The next morning, transfer both 5 mL starter cultures to a 1 L Erlenmeyer flask containing 250 mL of liquid LB with kanamycin (50 μg/mL final concentration).

d. Incubate at 28 °C with shaking (200 rpm) for 6–8 h until the OD_600_ reaches ~0.8.

*Note: After 4 h, monitor the OD_600_ frequently. See General note 11.*

e. Transfer the culture to a 250 mL Beckman tube and centrifuge for 10 min in a Beckman JA-14 Fixed-Angle Aluminum rotor (brake ON) at 6,574× *g*.

f. Discard the supernatant and resuspend the pellet in the appropriate volume of sucrose solution (see Recipes) to obtain a final OD_600_ of ~0.8.

*Note: Resuspend in 200 mL of sucrose solution and then adjust OD_600_ to 0.8.*

g. Check the final OD_600_ and adjust to 0.8.

h. Add 0.01% Silwet (0.1 μL/mL final solution) and mix well by inverting.

i. Pour all the solution into a 20–25 cm wide round container.

j. Submerge the inflorescences of 3–4-week-old *Arabidopsis* plants into the *A. tumefaciens*–sucrose solution for 10–20 s. Mix the solution while submerging to ensure all flowers get in contact with the *A. tumefaciens* solution.

**Critical:** Ensure that all unopened flowers are fully submerged to maximize transformation efficiency.

k. Cover the pot with a dark plastic bag or a tray to exclude light.

l. Leave the plants in darkness for 12–24 h.

m. Uncover the plants and water them.

n. Harvest seeds once plants reach senescence, typically 4–6 weeks post-transformation.

**Critical:** The drier the plant, the easier it is to collect seeds. See General note 9.

**4. Total RNA isolation from *Arabidopsis* inflorescences**

a. Collect 10–20 inflorescences of 4–6-week-old T1 plants into a 2 mL SafeSeal tube containing 4–8 Zirconia beads.

**Caution:** Liquid nitrogen can cause severe cryogenic burns; manipulate with protective gloves and glasses.

b. Snap freeze tubes in liquid nitrogen.

**Pause point:** Frozen inflorescences can be stored at -80 °C until use.

c. Grind the tissue in a bead beater.

*Note: If a bead beater is unavailable, grind the tissue in a cold porcelain mortar as described in steps E2–E3.*

**Caution:** The following steps involve using hazardous reagents; use protective gloves and glasses and work in a fume hood.

d. Add 1.3 mL of phenol/guanidine-based extraction buffer (see Recipes) and continue to grind the mixture until the sample is fully thawed and translucent.

e. Transfer the homogenate to a 2 mL tube.

f. Centrifuge at 4 °C for 5 min in an Eppendorf FA-24x2 rotor (brake ON) at 10,956× *g* to pellet cell debris.

g. Transfer the supernatant to a pre-chilled 2 mL tube.

h. Add 500 μL of cold chloroform and shake vigorously for 20 s.

i. Incubate at RT for 5 min.

j. Centrifuge at 4 °C for 5 min in an Eppendorf FA-24x2 rotor (brake ON) at 10,956× *g* to separate the phases.

**Critical:** Handle tubes gently to avoid mixing phase separation.

k. Use a 200 μL pipette to transfer the aqueous (upper) phase to a new pre-chilled 2 mL tube.

l. Add 1 mL of cold isopropanol and mix gently by inversion.

m. Incubate at RT for 10 min.

n. Centrifuge at 4 °C for 10 min in an Eppendorf FA-24x2 rotor (brake ON) at maximum speed to pellet RNA.

o. Carefully discard the isopropanol supernatant.

p. Wash the pellet with 300 μL of cold 75% ethanol (see Recipes).

q. Centrifuge at 4 °C for 1 min in an Eppendorf FA-24x2 rotor (brake ON) at maximum speed and remove the ethanol using a 1,000 μL pipette.

r. Briefly spin the tubes for 5 s and remove any residual ethanol using a 20 μL pipette.

s. Air-dry for 10 min.

t. Resuspend pellet in 50–100 μL of sterile Milli-Q water.

u. Transfer to a pre-chilled 1.5 mL tube and quantify RNA concentration using a NanoDrop.

v. Store at -80 °C until use.

**5. Real-time RT-qPCR**

a. Set up the DNase I reaction in a 1.5 mL tube as described in Table 9.

**Table 9.** DNAse I treatment reaction mix

Reagent	Volume
Total RNA	X μL for 2 μg
10× DNAse I buffer*	1.2 μL
DNAse I*	1 μL
Sterile Milli-Q water	Up to 12 μL

*From TURBO DNA-free Kit.

b. Incubate the reactions for 30 min at 37 °C.

c. Add 2 μL of inactivation buffer (from TURBO DNA-free Kit) and incubate for 5 min at RT.

*Note: It is recommended to vortex softly during the incubation time as the inactivation buffer tends to accumulate at the bottom of the tube.*

d. Centrifuge for 2 min in an Eppendorf FA-24x2 rotor (brake ON) at 5,590× *g*.

e. Transfer 9 μL of the supernatant to a new 1.5 mL tube and quantify RNA concentration with a NanoDrop.

f. Assemble the cDNA synthesis reaction in a 200 μL tube as described in Table 10.

**Table 10.** cDNA synthesis reaction mix

Reagent	Volume
DNAse I-treated Total RNA	X μL for 500 ng
5× PrimeScript buffer*	2 μL
Oligo dT primer*	0.5 μL
Random hexamers*	0.5 μL
PrimeScript RT enzyme mix I*	0.5 μL
Sterile Milli-Q water	Up to 10 μL

*From PrimeScript RT Reagent Kit.

g. Incubate the reactions under the following conditions (Table 11):

**Table 11.** Incubation times and temperatures to perform the cDNA synthesis reaction

Time	Temperature
15 min	37 °C
5 s	85 °C
Hold	4 °C

h. Transfer the tubes to ice and prepare a 1:5 dilution of each tube using sterile Milli-Q water.

i. Prepare the RT-qPCR reaction mix in a 96-well plate as shown in Table 12.

**Table 12.** Real-time quantitative PCR reaction mix

Reagent	Volume
TB Green Premix EX Taq*	10 μL
Forward primer	0.4 μL
Reverse primer	0.4 μL
ROX II reference dye*	0.4 μL
1:5 diluted cDNAs	2 μL
Sterile Milli-Q water	Up to 20 μL

*From TB Green Premix Ex Taq.

j. Set up the reaction steps as shown in Table 13.

**Table 13.** Steps of the quantitative PCR reaction

Time	Temperature	Phase
2 min	50 °C	Hold stage
10 min	95 °C	
15 s	95 °C	PCR stage
1 min	60 °C	
15 s	95 °C	Melt curve stage
1 min	60 °C	
15 s	95 °C	

k. To determine target mRNA accumulation from the data set generated after the qPCR reaction, see the Data analysis section. See General notes 12 and 13.

**E. Transgene-free virus-based syn-tasiRNA expression (Option II)**

**1. Agroinoculation**

a. Follow the instructions described in steps D1–D10 to obtain *A. tumefaciens* cultures transformed with the corresponding *pLBPVXBa-M-*based syn-tasiRNA construct.

b. Measure the OD_600_ and normalize cultures to a final OD_600_ of 0.5.

c. Infiltrate one patch in the central region of the fourth and fifth leaves (counting from the bottom) of 3–4-week-old *N. benthamiana* plants.

**Critical:** Place the inoculated plants to ensure they remain separated and their leaves do not contact.

**2. Preparation of crude extract and spray inoculation**

*Note: Plants start displaying PVX symptoms around 4–5 days post-inoculation (dpi), reaching a maximum between 6 and 8 dpi; then, symptoms start to disappear. PVX symptoms are characterized by a mild chlorotic mosaic, which is more evident in upper leaves.*

a. Collect 1–2 apical leaves (1 g) displaying PVX symptoms from each plant at 4–5 dpi.

**Caution:** Liquid nitrogen can cause severe cryogenic burns; manipulate with protective gloves and glasses.

b. Snap-freeze collected leaves in liquid nitrogen.

**Pause point:** Infected leaves can be stored at -80 °C until use.

**Critical:** Tissue must remain frozen throughout the grinding process. Pre-chilling the mortar and pestle with liquid nitrogen before crushing helps to prevent the sample from thawing.

**Caution:** The following steps involve using hazardous reagents; use protective gloves and glasses and work in a fume hood.

c. Grind the tissue with a porcelain mortar and pestle until obtaining a fine powder and homogenize it with 5 mL of cold inoculation buffer (see Recipes).

d. Use a 5 mL pipette with cut tips to filter the extract through a sterile Miracloth placed on top of a pre-chilled 50 mL conical tube.

e. Transfer the filtered extract to a pre-chilled 30 mL vaporizer.

**Figure 7.**
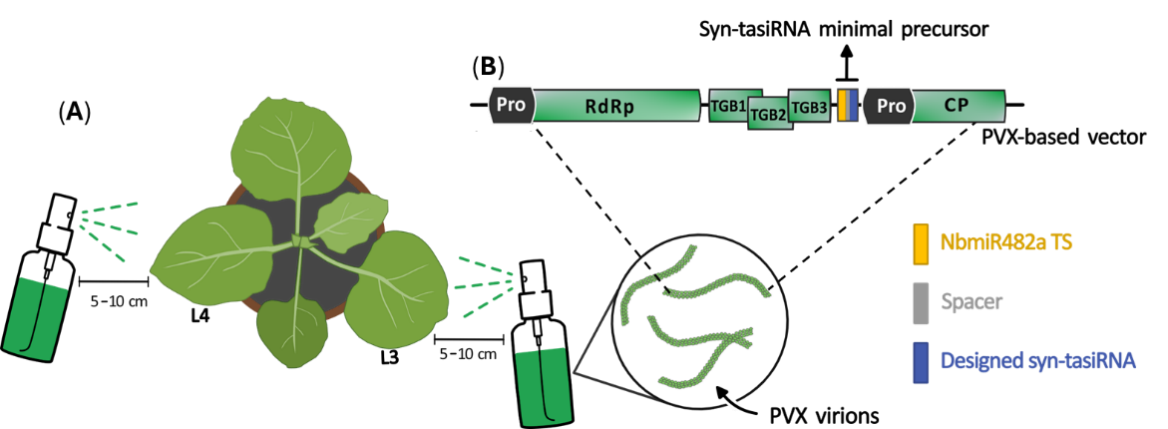
Spray application of crude extracts to *N. benthamiana* leaves. (A) Crude extracts obtained from virus-infected plants are sprayed into the third and fourth leaves (counting from the bottom, L3 and L4, respectively) of 3–4-week-old *N. benthamiana* plants from a distance of 5–10 cm using a vaporizer. (B) Viral particles present in the crude extract are produced following the expression of Potato virus X (PVX)-derived constructs. PVX open reading frames and promoters (Pro) are represented as light green and black boxes, respectively. The minimal syn-tasiRNA precursor is depicted by a grey box containing the NbmiR482a target site (TS; yellow box), a spacer (grey box), and a syn-tasiRNA sequence (blue box). RdRP: RNA-dependent RNA-polymerase. TGB: triple gene block. CP: coat protein.

**Critical:** After grinding, always keep the extract on ice until spray applications.

f. Spray the third and fourth leaves (counting from the bottom) of 3–4-week-old *N. benthamiana* plants at a 5–10 cm distance (Figure 7). Perform 2–4 applications to each leaf. After the application, a visible residue of carborundum remains on the surface of the leaf; do not rinse. See Figure S1 for visual details on the spraying procedure.

**Critical:** Shake the crude extract/vaporizer prior to each application.

**Critical:** Spray each plant isolated from the rest to avoid cross-contamination.

**Caution:** When spraying, use protective glasses, gloves, and a mask, and avoid contact of the extract with the skin. Spray in a well-ventilated room.

See Troubleshooting 6 and General note 14.

**3. Total RNA isolation**

a. Plants start displaying PVX symptoms of leaf curling around 4–5 days post-spraying (dps) and become more intense at 6–8 dps; then, they start fading. Later PVX symptoms are characterized by a mild chlorotic mosaic, which is more evident in upper leaves. Gene silencing phenotypes (if present) are mainly localized in upper leaves. Collect two apical leaves from each plant at 7–14 days after the spray inoculation.

**Caution:** Liquid nitrogen can cause severe cryogenic burns; manipulate with protective gloves and glasses.

b. Snap-freeze leaves in liquid nitrogen.

**Pause point:** Infected leaves can be stored at -80 °C until use.

c. Follow steps E2–E19.

**4. Real-time RT-qPCR**

a. Follow the steps described in Section H.

## Data analysis

For the analysis of the data generated from the qPCR reaction (Section H):

1. For statistical robustness, it is recommended to infiltrate/inoculate at least three *N. benthamiana* plants per syn-tasiRNA construct.

2. To assess pipetting accuracy, each biological replicate is measured in duplicate (technical replicates).

3. Target mRNA accumulation is calculated using the comparative delta cycle threshold (C_T_) method (ΔΔC_T_), which compares the normalized C_T_ (ΔC_T_) of the sample to that of a reference gene.

4. The ΔΔC_T_ is calculated using the arithmetic mean of all measurements from a biological block, which contains both technical replicates for each biological replicate.

5. The relative expression level is then determined as 2^^ΔCT^.

6. The copy number of each sample is represented relative to a control sample, which is typically a plant agroinfiltrated/agroinoculated with the *pMDC32-GUS* construct.

## Validation of protocol

This protocol has been entirely validated in the following research article:

• Cisneros et al. [4]. Syn-tasiR-VIGS: virus-based targeted RNAi in plants by synthetic trans-acting small interfering RNAs derived from minimal precursors. *Nucleic Acids Research*, 2025.

In this article, minimal syn-tasiRNA precursor validation in *Arabidopsis* is shown in Figure 1D, displaying results from plants expressing minimal syn-tasiRNA precursors targeting two different endogenous genes. The ability to express several syn-tasiRNAs simultaneously from a minimal precursor is validated in Figure 2E. On the other hand, the use of the minimal syn-tasiRNA precursor in *N. benthamiana* is validated in Figure 4B, targeting an endogenous gene. In all cases described above, syn-tasiRNA silencing efficiency is validated by RT-qPCR performed over three independent biological replicates. The control sample in each case is the minimal syn-tasiRNA construct targeting *E. coli*’s β-glucuronidase *GUS* gene, which acts as a negative control. Statistical differences are determined by a pairwise Student’s *t*-test, with p < 0.05 being significantly different.

Validation of the transgene-free approach in *N. benthamiana* is represented in Figure 7B and C, where an endogenous gene is targeted. In this case, data is validated by RT-PCR detection of syn-tasiRNA minimal precursor and PVX genome in three independent biological replicates. Finally, the transgene-free approach is also validated by targeting an exogenous gene in a multiplexed precursor, ultimately conferring resistance against a pathogenic virus (Figure 7D and E). Plant viral resistance is also validated by determining viral protein accumulation by western blot in three independent biological replicates (Figure 7E).

## General notes and troubleshooting

**General notes**

1. Gene silencing efficiency of the syn-tasiRNA-based construct could vary between plant species, genes, and target regions. Therefore, it is highly recommended to test the individual silencing efficiency of different syn-tasiRNAs designed to distinct target regions in the species of interest, prior to assembling a multiplexed syn-tasiRNA construct.

2. When aiming to achieve antiviral resistance using a combination of syn-tasiRNAs, it is recommended to first test the antiviral activity of each art-sRNA individually. Once identified, the art-sRNAs with the highest antiviral activity are combined in a single syn-tasiRNA construct. A specific protocol for a fast-forward identification of art-sRNAs with high antiviral activity is provided here [7].

3. When expressing multiple syn-tasiRNAs (up to four syn-tasiRNAs can be efficiently expressed simultaneously from the same precursor), keep in mind that those positioned farther from the miRNA TS tend to accumulate less in vivo, resulting in weaker target gene silencing. This positional effect can be used to fine-tune gene expression. A detailed protocol on how to apply this strategy can be found here [8].

4. Other tools that could be used to design art-sRNAs are WMD3 (http://wmd3.weigelworld.org/cgi-bin/webapp.cgi) [9] and pssRNAit [10].

5. In vivo off-target effects of syn-tasiRNAs can be assessed, for example, by combining genome-wide transcriptome profiling with 5′ RNA ligase-mediated rapid amplification of cDNA ends (5′RLM-RACE, as done before for amiRNAs [11]).

6. An alternative way to generate the *pLBPVXBa-M-*based construct is to amplify the syn-tasiRNA minimal precursor from a previously generated *pMDC32B*-based construct using the oligonucleotides below (Table 14). The resulting PCR product can then be cloned into *pLBPVXBa-M* following the steps detailed in Section B.

**Table 14.** Oligonucleotide sequence to amplify the minimal syn-tasiRNA cassette.

Oligonucleotide	5′ to 3′ DNA sequence
Forward	AGAGGTCAGCACCAGCTAGC + miRNA TS sequence (22 nt)
Reverse	AGGGAAACTTAACAAACCCT + reverse-complementary last syn-tasiRNA sequence (21 nt)

7. Sow *N. benthamiana* seeds in a seedbed. After 1 week, transplant four plants into a 15-cm pot. Grow plants in a growth chamber at 25 °C with a 12/12 h light/dark photoperiod. Typically, three plants are used from each pot after 3–4 weeks.

8. If the targeted gene induces a visible phenotype when silenced, it is recommended to infiltrate small patches in a few leaves of a second batch of plants to easily evaluate silencing efficiency.

9. Depending on the number of infiltrated leaves, the final culture volume can be scaled up or down, as long as the final OD_600_ is maintained. This also applies to Section F.

10. Sow *Arabidopsis* Col-0 seeds in a seedbed. After 1 week, transplant 10–12 plants into a 15-cm pot. Grow plants in a growth chamber at 22 °C with a 16/8 h light/dark photoperiod. Use 4–6 pots per construct for floral dipping.

11. To reduce the number of wild-type seeds, it is helpful to remove all siliques and open flowers from 3–4-week-old *Arabidopsis* plants prior to transformation. This step can be performed while the culture is growing.

12. Target mRNA levels are measured relative to a reference gene, such as *NbPP2A* and *AtACT2* for *N. benthamiana* and *Arabidopsis*, respectively. Use the following oligonucleotides for RT-qPCR when using *NbPP2A* or AtACT2 as the reference gene (Table 15).

**Table 15.** Oligonucleotide sequences for reference gene amplification

Oligonucleotide	5′ to 3′ DNA sequence
*NbPP2A*-Forward	GACCCTGATGTTGATGTTCGCT
*NbPP2A*-Reverse	GAGGGATTTGAAGAGAGATTTC
*AtACT2*-Forward	AAAAATGGCTGAGGCTGATGA
*AtACT2*-Reverse	GAAAAACAGCCCTGGGAGC

13. Syn-tasiRNA production can be determined through several techniques. Detection of sRNAs by Northern blot is one option; a specific protocol is provided here [12]. Detection of the syn-tasiRNA precursor is another option, which can be performed by RT-PCR to determine its presence in treated tissues.

14. To confirm PVX infection in agroinoculated (Section I) and sprayed plants (Section J), perform PCR over cDNA samples generated from collected tissue (steps described in Sections E and H) with the oligonucleotides in Table 16. In samples from infected plants, a 627 bp band will be amplified, which will be absent in non-infected plants.

**Table 16.** Oligonucleotide sequences for PVX detection in infected plants.

Oligonucleotide	5′ to 3′ DNA sequence
PVX-Forward	ATGTCAGGCCTGTTCACTATCC
PVX-Reverse	TGGTGGTGGTAGAGTGACAAC

**Troubleshooting**

Problem 1: No or low *E. coli* colonies are obtained after transformation.

Possible cause: The reaction contains impurities or is too diluted.

Solution: Purify the digestion-ligation reaction using a nucleic acid purification column such as Zymo spin columns.

Problem 2: Insufficient amount of linearized plasmid for Gibson assembly.

Possible cause: The circular plasmid concentration is too low, or the digestion was incomplete.

Solution: Digest a larger amount of plasmid, perform multiple parallel digestions, and purify them together. Extend the digestion time to ensure complete linearization.

Problem 3: High proportion of empty clones after Gibson assembly.

Possible cause 1: The vector was not completely linearized.

Solution: Refer to the solution for Problem 2.

Possible cause 2: The insert/vector ratio is too low.

Solution: Make sure to add the proper amount of insert indicated by the manufacturer, considering the insert length.

Problem 4: The starter culture is not saturated after 24 h.

Possible cause: The plate used to inoculate the culture was too old.

Solution: Use a fresh plate (1–3 days old) to initiate the starter culture.

Problem 5: Leaves do not take up the infiltration volume.

Possible cause: Leaves are too rigid or turgid (contain excess water).

Solution: Avoid watering plants on the day of infiltration. If the problem persists, puncture the leaf with a 1 μL pipette tip and infiltrate through the orifice.

Problem 6. No symptom appearance or low rate of infection in sprayed *N. benthamiana* plants.

Possible cause 1: Low PVX concentration in the crude extracts.

Solution: Symptom detection and leaf collection times in agroinoculated plants are critical to ensure a highly infectious crude extract. If the problem persists after meticulously following protocol steps and collection times, consider increasing the amount of tissue collected from agroinoculated plants to 2–3 g. If doing so, make sure to scale up the rest of the protocol volumes.

Possible cause 2: Incorrect handling of crude extract.

Solution: Make sure the extract is constantly on ice and reduce to a minimum the time between crude extract generation and spray application. Increase the number of spray applications in each leaf.

## Supplementary information

The following supporting information can be downloaded here:

1. Protocol S1. Preparation of *E. coli* (strain DH5*α*) electrocompetent cells.

2. Protocol S2. Preparation of *A. tumefaciens* (GV3101 strain) electrocompetent cells.

3. Figure S1. Spray inoculation of crude extracts on *N. benthamiana* plants.
